# Development of stochastically reconstructed 3D porous media micromodels using additive manufacturing: numerical and experimental validation

**DOI:** 10.1038/s41598-024-60075-w

**Published:** 2024-04-23

**Authors:** Dongwon Lee, Matthias Ruf, Nikolaos Karadimitriou, Holger Steeb, Mary Manousidaki, Emmanouil A. Varouchakis, Stelios Tzortzakis, Andreas Yiotis

**Affiliations:** 1 Institute of Applied Mechanics (CE), University of Stuttgart, Pfaffenwaldring 7, 70569 Stuttgart, Germany; 2 SC SimTech, University of Stuttgart, Pfaffenwaldring 5, 70569 Stuttgart, Germany; 3 Institute of Electronic Structure and Laser, Foundation for Research and Technology-Hellas, 71110 Heraklion, Greece; 4 School of Mineral Resources Engineering, Technical University of Crete, 73100 Chania, Greece

**Keywords:** Chemical engineering, Civil engineering

## Abstract

We propose an integrated methodology for the design and fabrication of 3D micromodels that are suitable for the pore-scale study of transport processes in macroporous materials. The micromodels, that bear the pore-scale characteristics of sandstone, such as porosity, mean pore size, etc, are designed following a stochastic reconstruction algorithm that allows for fine-tuning the porosity and the correlation length of the spatial distribution of the solid material. We then construct a series of 3D micromodels at very fine resolution (i.e. $16\,\upmu$m) using a state-of-the-art 3D printing infrastructure, specifically a ProJet MJP3600 3D printer, that utilizes the Material Jetting technology. Within the technical constraints of the 3D printer resolution, the fabricated micromodels represent scaled-up replicas of natural sandstones, that are suitable for the study of the scaling between the permeability, the porosity and the mean pore size. The REV- and pore-scale characteristics of the resulting physical micromodels are recovered using a combination of X-ray micro-CT and microfluidic studies. The experimental results are then compared with single-phase flow simulations at pore-scale and geostatistic models in order to determine the effects of the design parameters on the intrinsic permeability and the spatial correlation of the velocity profile. Our numerical and experimental measurements reveal an excellent match between the properties of the designed and fabricated 3D domains, thus demonstrating the robustness of the proposed methodology for the construction of 3D micromodels with fine-tuned and well-controlled pore-scale characteristics. Furthermore, a pore-scale numerical study over a wider range of 3D digital domain realizations reveals a very good match of the measured permeabilities with the predictions of the Kozeny–Carman formulation based on a single control parameter, $k_0$, that is found to have a practically constant value for porosities $\phi \ge 0.2$. This, in turn, enables us to customize the sample size to meet REV constraints, including enlarging pore morphology while considering the Reynolds number. It is also found that at lower porosities there is a significant increase in the fraction of the non-percolating pores, thus leading to different $k_0$, as the porosity approaches a numerically determined critical porosity value, $\phi _c$, where the domain is no longer percolating.

## Introduction

Transport processes in geologic macroporous media are relevant to diverse and ubiquitous environmental and energy-related applications, ranging from geothermal and water aquifer flows to oil recovery, and CO_2_ sequestration in deep geological formations. Such processes have been traditionally modeled based on a unified field-scale approach, where the porous medium is treated as an effective continuum [the so-called Representative Elementary Volume (REV)], while momentum, mass and heat transport are considered to be proportional to the gradients of pressure, species concentration and temperature, respectively^1–3^. Under such an approach, the pore-scale heterogeneities, that appear at smaller length scales, are expressed and quantified through lumped transport properties (defined at the REV scale), like the intrinsic permeability of the medium and dispersion coefficients for flow and mass transport, respectively. Such an approach allows for the efficient (for engineering purposes) modeling of the complex large scale systems commonly involved in geologic flows, at the cost, however, of a physically consistent description of pore-scale effects, even in typical flow configurations.

Immiscible two-phase flow in porous formations is a classic example of a process where the established continuum approach often fails to appropriately describe flow dynamics in a physically consistent manner under specific flow conditions. In this case, the movement of interfaces through the porous matrix gives rise to highly non-linear phenomena, such as viscous fingering^4,5^, that significantly affect the sweeping efficiency of the invading phase at the REV-scale due to physical interactions emanating at the pore-scale. Due to such interactions, the percolating front (for the specific case of a fixed pair of fluids) may vary from practically flat (stable), to completely unstable (viscosity-controlled) under different flow conditions. Such transitions cannot be adequately modeled by the classic relative permeability formulation, where the later is assumed to be exclusively a function of the local phase saturation^6–8^.

Significant insight on the effects of pore-scale interactions on the field-scale behavior of such non-linear processes (such as the dynamics of interfaces in immiscible flows, or the hydrodynamic dispersion of dissolved species) has been provided by more elaborate studies using simplified, and largely exaggerated in size, transparent micromodels with porous-like structures. These micromodels, commonly known as pore-network or pore-scale models, allowed for the direct visual observation of transport phenomena under very well-controlled flow conditions, thus offering a better understanding of the correlation between pore- and field-scale dynamics, which is otherwise masked by the opaque nature of real porous media, coupled with the very small length scale where these phenomena emanate. Such studies using transparent predominantly 2D micromodels have revealed important, and previously unknown effects, such as the flow of the non-wetting phase in the form of discontinuous ganglia under the sweeping effects of a continuous wetting phase^9^. They have thus served as the basis for the development of more complex field-scale numerical models where the relative permeability was formulated as a second order tensor with non-zero off-diagonal terms in order to accommodate viscous coupling and momentum transfer across the interfaces^10^.

Experimental studies using 2D pore-scale models have also provided valuable insight on the effects of the competition between viscous and capillary forces on the dynamics of the interfaces during also immiscible two-phase flows. In a seminal contribution, Lenormand and coworkers^11^ have demonstrated the combined effects of the fluid viscosity ratio and the Capillary number of the flow on the evolving field-scale shape of the interfaces. The phase-map developed in this study has been since used for the interpretation of different flow regimes observed in a series of subsequent studies^12^. More recently, in a series of contributions using transparent 2D micromodels, Tallakstad et al.^13^ and Chevalier et al.^14^ have demonstrated a divergence from the established Darcy-scale regime during the mobilization of stranded ganglia at intermediate Capillary numbers. These contributions also highlighted the effects of flow history in steady state relative permeability values besides the common dependence on the saturation.

Besides their invaluable contribution to the understanding of the effects of pore-scale physics on the field scale dynamics, experimental approaches based on transparent 2D micromodels lack two key characteristics of natural porous materials; a) a physically consistent three-dimensional structure, and b) a realistic representation of the pore-scale heterogeneity pertinent to natural geologic porous media. In recent years, the development of novel additive manufacturing, or 3D printing, methodologies has allowed for the construction of 3D porous micromodels^15–19^, as the means to obtain better insight into the relevant physical phenomena without the above simplifications. This approach offers additional advantages, as it ensures sample reproducibility and allows for customized specimen design to test specific hypotheses. It enables researchers to conduct numerical and experimental studies in order to identify dominant physical mechanisms, while systematically adjusting intended properties, such as porosity or pore throat sizes, as needed. In contrast, natural rock samples present challenges in these investigations due to their inherent heterogeneity, anisotropy and natural variability. While these early contributions proposed rather simplistic 3D porous structures with little structural resemblance to naturally-occurring porous material, such approaches allowed for the fast-prototyping of 3D micromodels based on complex algorithms for the spatial distribution of the solid material^20,21^. By combining experimental and numerical results the optimization of the pore geometry itself can be achieved, on demand^22,23^.

Additive manufacturing is a process of producing micromodels in a layer-like manner based on a variety of different technological approaches. The most widespread of such technologies rely on material extrusion, vat photopolymerization, material jetting, powder bed fusion, binder jetting, among many others^24^. In material extrusion, the printer extrudes a molten thermoplastic, which behaves as a highly viscous fluid, and deposits it layer by layer following a digital design, thus forming a 3D structure. In vat photopolymerization, the printing platform contains a tub filled with a resin which can be cured with typically ultraviolet (UV) light. By selectively exposing the photosensitive resin to UV light, the resin shape changes and solidifies according to the designed pattern by the user. In material jetting, also referred to as MultiJet printing technology, a printing nozzle deposits droplets of photopolymer plastic on a plate, which are then instantly cured by UV light. It is different than the vat approach, since material jetting does not require a tub of resin. Due to the absence of this constriction of using a tub with the material, it is possible to use several printing materials. Commonly, a support material is also used in order to prevent the mechanical failure of the structurally weak part. The supporting material can be, and is, removed after printing^25^. In powder bed fusion, the method is based on the use of a tub of printing material powder. A thermoplastic, glass beads or even metals can be used as the printing material. By selectively sintering, or even melting, the corresponding material, the powders bond, and a solid 3D structure can be formed layer by layer. In the powder bed fusion method, lasers are typically used as a heat source^26^. The Binder jetting method also utilizes a bed of powder similar to powder bed fusion. The difference is that binder jetting uses adhesive to bond particles, something which offers a variety in the available printing materials. By depositing the adhesive in a designed pattern layer by layer, a final 3D structure can be obtained^27^.

In recent years, several of the above described additive manufacturing approaches have been used for the construction of the pore space geometry of three-dimensional, realistic porous media as summarized in Table 1. Such studies mostly focus on the macroscopic properties of porous media, such as the porosity-permeability correlation^20,21^, and the elastic properties of un-/saturated samples of natural rock proxies^28^ . Huang et al.^28^ used Fused Deposition Modeling (FDM) technology with a Stratasys Dimension SST 768 printer to fabricate a cube-shaped porous medium measuring 25 mm on each side. FDM is a 3D printing method that melts a plastic filament and deposits it layer by layer to create a 3D object. Due to the layer-by-layer deposition of FDM, the fabricated sample contains innate default porosity between layers where each layer is of a plane of filament. In order to mimic cracks in a real porous medium, they incorporated a regular pattern of penny-shaped pores in their sample. They conducted mechanical loading tests on both saturated and unsaturated samples and found a good agreement with the theoretical value, as determined by following Hudson’s equation^29^. However, the default porosity imposed by the nature of FDM was embedded without any control, and neither its connectivity nor isotropy was evaluated, even though these parameters can significantly affect the reported results.

Head and Vanorio^21^ studied numerically and experimentally the permeability of rock microstructures with the help of a 3D printed, single pore structure. For the fabrication of their samples, they used 3Dsystem ProJetHD 3000 Plus, which employs a multijet technique at a resolution of 16 $\mu$m. By using XRCT, the single pore geometry was obtained from a natural carbonate sample. The obtained geometry was either isotropically (scaled) or anisotropically deformed (pore throat enlargement) in order to replicate the compacted and dissolved pore spaces found in natural specimens. Those combinations of transformed geometries were fabricated. Subsequently, permeability measurements were performed experimentally and numerically, based on those samples. Their results enhanced our insight into the power-law-based porosity-permeability relation for compaction and dissolution, by proposing a fitting coefficient for each case.

In the work of Li et al.^20^, the authors demonstrated their numerical and experimental investigation of fluid transport in artificial, discrete fracture networks. The cylindrically-shaped samples were fabricated by using the Objet500 Connex multijet 3D printer at a resolution of 16 um. The size of samples was 50 mm in diameter and 100 mm in height. Each sample contained fracture(s) with a designed surface roughness, with the mean aperture of the fractures being 500 $\mu$m. Based on these samples, a parametric study was performed to explore the effects of the number of fractures and surface roughness on permeability. According to their findings, the critical hydraulic gradient that marks the transition from linear to non-linear flow, where the linear relation between flow rate and pressure difference no longer holds, decreased as the aperture, roughness, and number of fractures increased. Additionally, in order to validate the geometry precision of the printed samples, they performed numerical investigations based on the XRCT-characterized geometries of the printed samples. Their validation results showed that the precision of the printed sample decreased when the sample contained more fractures with more intersections.

More recently, the 3D binderJet technique has been adopted in order to produce proxies of natural rock samples^30–32^. Since the printing method utilizes granules (or powder) and binds them with adhesives, one can fabricate a specimen which contains designed macro pores and micro pores which are shaped by the shape of the granules. Unlike previously mentioned stereolithographic printing methods, this approach could provide a great potential to resolve very fine micro pores while producing final outputs in relatively bigger scales.

In the study of Ishutov et al.^33^, the authors investigated a proxy which was created by using ProJet 660 Plus 3D printer with the binder jet technique. They used gypsum powder as their printing material. Since the resolution of the printer was 150 um while the mean pore throat of the target (Fontainebleau sandstone) was 30 $\upmu$m^34^, they fabricated their replica after upscaling the obtained geometry with the use of XRCT scans. The fabricated samples demonstrated a good match in terms of permeability and pore throat size distribution, indicating that 3D printed samples have great potential to be used in the production of rock replicas. However, a significant variation in porosity was mainly induced during post-processing with compressed air.

Song et al.^31^ manufactured rock analogues with three different materials, such as silica sand and gypsum powder, using binder jet technology, and coated silica beads using selective laser curing. The samples consisting of silica sand and gypsum powder were fabricated by a VX1000 3D printer at 200 $\mu$m resolution, while the coated silica beads sample was created by EOS PA 2200 at 100 um resolution. The samples had a cylindrical shape without giving any internal structure input. Through tomographic visualization methods (SEM and XRCT), the average pore and pore throat sizes were evaluated and compared with the natural Berea sandstone. Their results showed that the manufactured samples had higher porosity, pore and pore throat sizes compared to Berea sandstone (coated silica beads : 38.2 %, silica sand : 47.47 %). Interestingly, the pore size investigation of gypsum sample was missing in their study since its particle size was too small to be resolved with XRCT. Based on a subsequently performed numerical study with pore network model, the corresponding permeabilities of the printed samples were also 3 to 2.5 times higher than the natural ones.

A significant technological bottleneck reported in all previously cited works is related to the printing resolution which is required to efficiently reproduce realistic 3D proxies of geologic porous media. Due to this limiting factor, the experimental replicas fabricated by 3D printers typically diverge to some extent from the intended design at smaller length scales. This effect induces significant deviations between the absolute truth, meaning the natural porous medium, and the experimental and numerical results^35,36^. Such deviations can also have a strong influence on the printed geometries, potentially change the mechanical and overall properties of the printed sample in comparison to the actual one^37–39^. Although many efforts have been made in order to narrow this gap by improving the printing procedure^40,41^ or by choosing different printing materials^42,43^ for various printing methods, recent studies have shown that room for improvement still exists^30,44,45^. In the case of rock studies, this problem is complementary to the fact that occasionally the pore size distribution of natural rocks includes pore sizes which cannot be achieved with 3D printing, as they can be pretty small^17,46,47^. In recent works, the studies which were performed with binder jet technology showed a promising result in terms of resolving micro structures of natural rocks due to its inherent granular utilizing approach^33,48^. However, the limitation was also revealed that the inner structures were fabricated without control thus, making it very challenging to narrow the the gap between the natural rock and the printed sample.

As a compromise between the achievable resolution and the dimensionality of a natural porous medium, modern studies adopted an approach where they magnify the geometry of a natural rock to meet the resolution limitation of their printers^17,31,49^. However, this approach requires a careful study on the representativeness of the domain since the extracted domain could be too specific or broad to capture a scientific interest.

By taking these issues into account, the necessity for validation of printed samples rises in two perspectives; (1) validation of printed geometry, (2) representativeness of the printed sample. These validations could be performed via the combination of numerical and experimental investigations with the printed samples. As it has already been shown^20,21,31,33^, the printed 3D geometry could be acquired by using a tomographic approach such as XRCT and/or SEM. A difference between the design and the printed sample, or defects, for example, a deviation in terms of porosity or unintended cracks induced during post-processing of printing, can be directly detected. As a means of validation, a quantification via numerical and experimental investigation, for instance, permeability estimation, would be preferable since this can provide further insight into flaws and deviations^20,21,31,48^ on an REV scale. In addition, after the validation, the representativeness of the sample could be evaluated via subsequent numerical investigation on its domain size.

In this direction, we present in this study an integrated workflow for the digital design, additive manufacturing and characterization of realistic 3D micromodels that bear the pore-scale characteristics of sandstone , with a focus on single-phase flow during characterization. Despite the fact that these micromodels correspond to scaled-up replicas of naturally occurring porous media (due to technological limitations), they can serve for the study of single flows, and especially the effects of pore-scale characteristics (that are tunable in our approach) on the permeability and other transport properties, as long as the corresponding dimensionless number, e.g. Reynolds number, is respected. We provide evidence of the suitability of these micromodels for investigating the impact of pore-scale microstructure on the field-/REV-scale characteristics of the samples by employing advanced techniques, including state-of-the-art X-Ray Computed Tomography (XRCT) imaging, microfluidic measurements and robust numerical modeling at sub-pore-scale resolutions.

**Table 1:** Summary of additive manufacturing approaches for the fabrication of porous micromodels.

Authors	Dim	Printing methods	Printer (provider)	Resolution ($\upmu$m)	Domain size (mm)	Target	Numerical study
Watson et al.	2D	Stereolitography	Formlabs Form2	x: 25, y: 50, z: 100	(Cuboid-xyz) 20 $\times$ 10 $\times$ 0.3	Flow channels	Navier–Stokes
Ahkami et al.	2D	MultiJet	3D-labs GmbH	z: 75	(Cuboid-xyz) 1000 $\times$ 850 $\times$ 0.3	Fractured porous media	Lattice-Boltzmann
Huang et al.	3D	Fused deosition modeling	Stratasys dimension SST 768	z: 254	(Cube-xyz) 25 $\times$ 25 $\times$ 25	Porous media	–
Head and Vonorio	3D	Stereolithography, MultiJet	Formlabs Form1, 3D system	25, 16	(Cylindrical-dh) d: 2.4 to 3.4, h: 2.8 to 5	Single pore	Analytic, lattice-Boltzmann
Li et al.	3D	MultiJet	Object5000 Connex	16	(Cylindrical-dh) d: 50, h: 100	Fractures	Navier–Stokes
Ishutov et al.	3D	BinderJet	Projet 660 Plus	150	(Cube-xyz) 14.5 $\times$ 14.5 $\times$ 14.5, 43.5 $\times$ 43.5 $\times$ 43.5	Rock proxy	Swanson’s equation
Kong et al.	3D	MultiJet	Projet 460 Plus	200	(Cylindrical-dh) d: 50, h: 120, d: 25, h: 60	Rock proxy	- -
Song et al.	3D	Selective laser curing, BinderJet	EOS PA 2200, VoxelJet 1000	z: 100, z: 200	(Cylindrical-dh) d: 25, h: 50	Rock proxy	Pore network model

## Methodologies for 3D micromodel design and numerical characterization

### Stochastic reconstruction of digital porous domains

The design of the 3D porous structures developed in this study is based on the stochastic reconstruction algorithm proposed by Quiblier^50^, Adler et al.^51^ and Hyman et al.^52^. Each digital domain is constructed by first producing a cubic domain of size $L^3=500^3\,\delta x^3$ of random real numbers in the open interval (0,1) selected from a uniform distribution. Then, a 3D discrete Fourier transformation is computed on the domain data and multiplied by a Gaussian function, as follows;

1 $$\begin{aligned} Z'(\vec {k})= Z(\vec {k}) e^{-\frac{\left| \vec {k} \right| ^2}{\lambda ^2_0}} \end{aligned}$$

where $\vec {k}$ is a position vector and $\lambda _0$ a parameter related to the desired correlation length, $\lambda _s$ (i.e. a measure of the pore diameter in lattice units), as $\lambda _0=L/\lambda _s$

The inverse fast Fourier transformation is then performed;

2 $$\begin{aligned} f(\vec {r})=F^{-1}_T(Z'(\vec {k})) \end{aligned}$$

This eventually leads to a correlated and periodic 3D field of real values, $f(\vec {r})$, where the initial random noise $W(\vec {r})$ now follows the auto-correlation function;

3 $$\begin{aligned} (f*f)(x)\propto e^{-\frac{{\lambda ^2_0}}{8}x^2} \end{aligned}$$

This means that the domain values are now correlated in 3D space over distances proportional to $\lambda _s$. This parameter is considered as a proxy for the typical pore size^53^ and it thus represents the pore-scale resolution.

The binary domains are produced by applying a threshold value for all the domain voxels in order to recover a pre-defined porosity value $\phi$. This is achieved efficiently by calculating the cumulative distribution function *P*(*f*) for all voxel values $f(\vec {r})$ and thus obtaining the threshold value $f_0=P^{-1}(\phi )$ that satisfies the required porosity. Then, all positions with values below $f_0$ are taken as solid and the remaining voxels as void. The resulting domain is then binary (i.e. 0 for void and 1 for solid voxels) and periodic in all three spatial directions. More details regarding this approach are reported in Yiotis et al^54^.

**Figure 1:**
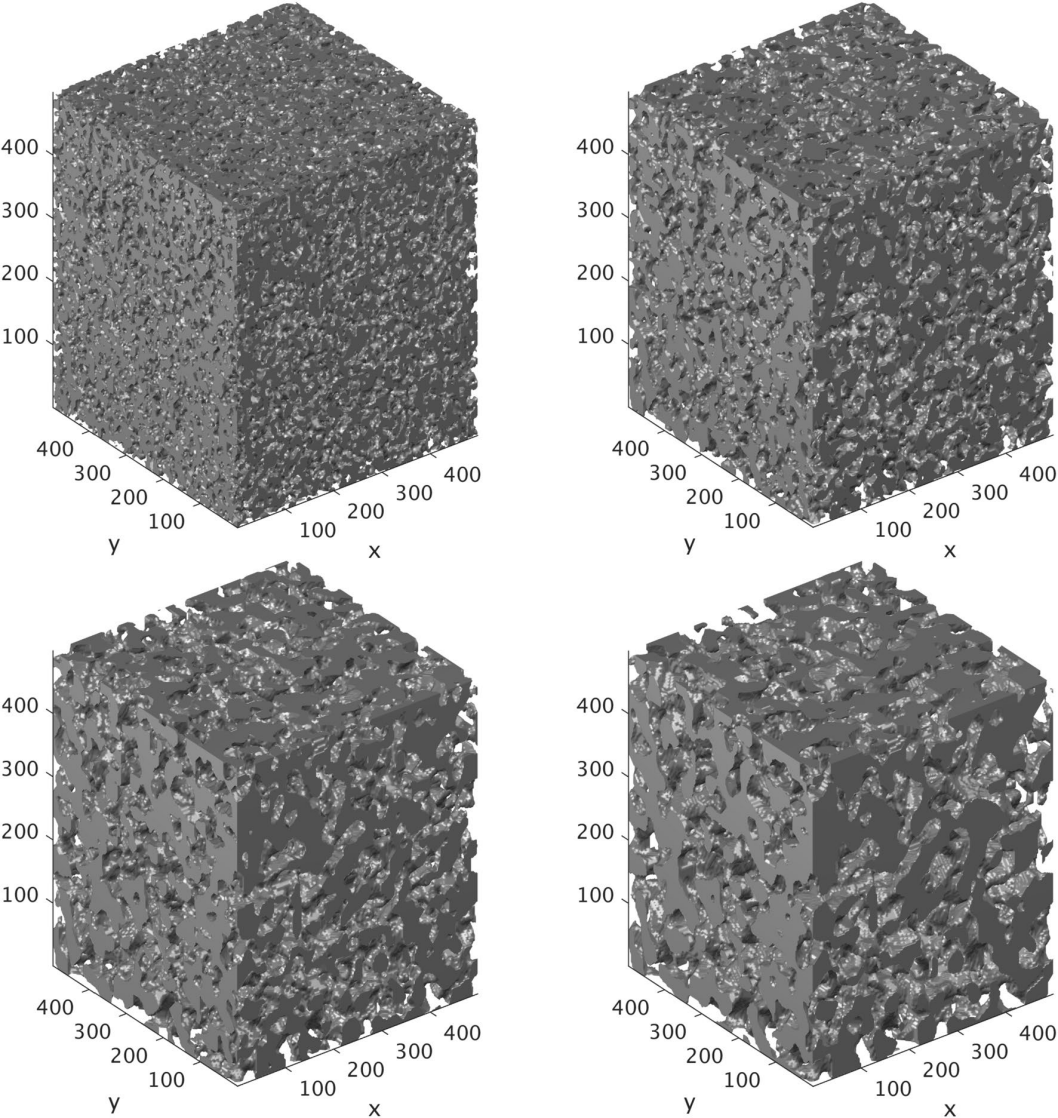
Stochastically reconstructed periodic 3D porous domains for different values of $\lambda _s$ and $\phi =0.45$, $L=500\delta x$. (top left) $\lambda _s=15\delta x$, (top right) $\lambda _s=25\delta x$, (bottom left) $\lambda _s=35\delta x$, and (bottom right) $\lambda _s=45\delta x$.

Characteristic snapshots of the resulting cubic porous domains are shown in Fig.1. These domains have exactly the same porosity, $\phi$=0.45, but different correlation lengths, $\lambda _s$, in the range of $15\,\delta _x \le \lambda _s \le 45 \,\delta _x$. Apparently, lower values of $\lambda _s$ produce smaller pores (e.g. top left), while larger values result in much larger sizes (e.g. bottom right). It should be also expected that the number of pores in these domains of fixed size *L* decreases with $\lambda _s$, whilst their resolution increases. The latter effect has an impact on the accuracy of the numerical approaches applied at sub-pore-scale resolution in order to recover the properties of the effective continuum (e.g. permeability), as will be also demonstrated below.

**Figure 2:**
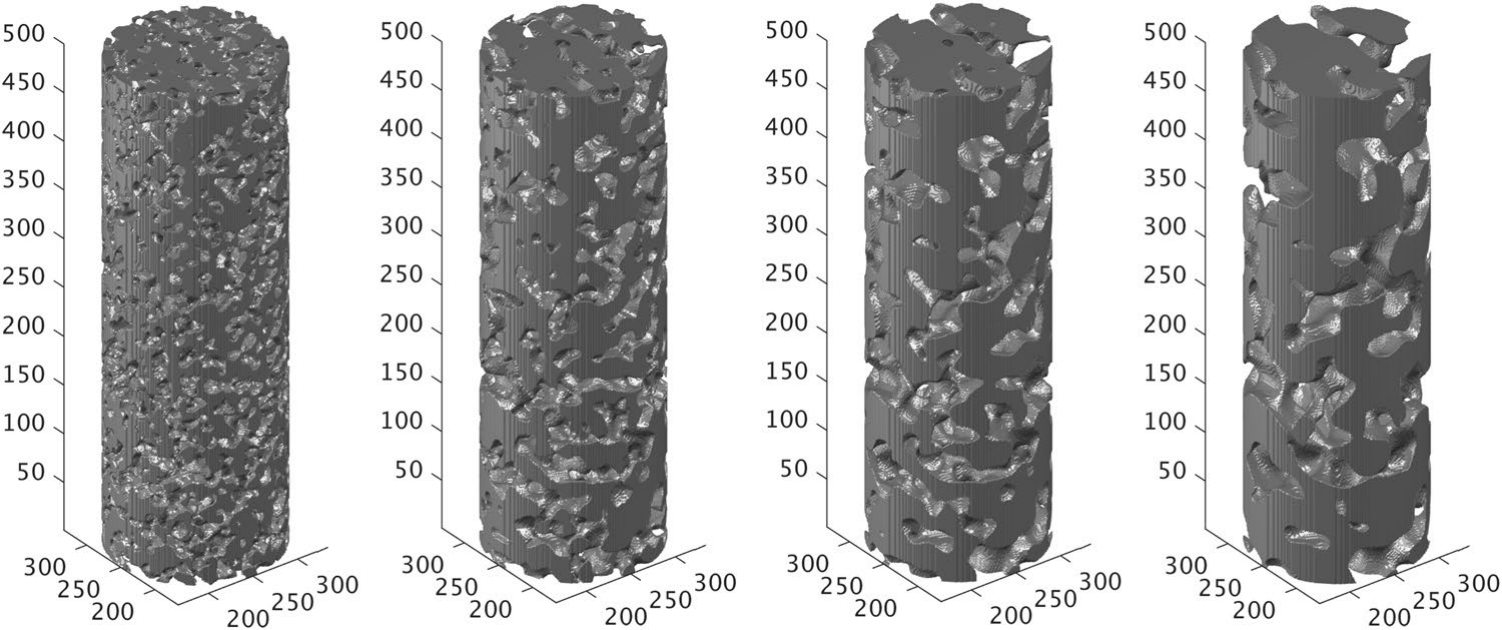
Cylindrical domains of height $L=500\,\delta x$ and diameter $D_0=L/3$ extracted from the cubic domains shown in Fig. 1. (From left to right) $\lambda _s=15\,\delta x$, $\lambda _s=25\,\delta x$, $\lambda _s=35\delta x$, and $\lambda _s=45\,\delta x$.

The final digital domain that will be converted to the physical 3D micromodels is a cylindrical section of height $L=500\,\delta x$, and is extracted from the central region of the previously generated cubes. This is generated by assigning a zero (void) value to all voxels of the initial cubic domains where the following condition is satisfied;

4 $$\begin{aligned} d=\frac{\left| {(\vec {r}-\vec {r_1}) \cdot (\vec {r}-\vec {r_2})} \right| }{ \left| \vec {r_2}-\vec {r_1} \right| } \ge R \end{aligned}$$

where $\vec (r_1)=(L/2,L/2,1)$, $\vec (r_2)=(L/2,L/2,L)$ and $R=L/6$. This produces a porous domain with a height of $L=500\,\delta x$ and a diameter $D=2R=166\,\delta x$ (see Fig. 2) that will serve as the conceptual design for the 3D micromodels employed in our study. It should be also noted that these domains remain periodic only in the z-direction (vertically). Furthermore, the cylindrical domains contain much less pores than their “parent” cubic ones. It should thus be expected that the cylindrical domains may no longer be representative of the original designs and cannot be considered as Representative Elementary Volumes (REVs). We will address this issue both numerically and experimentally in the following sections.

### Numerical characterization at the pore and REV scales

The 3D digital porous domains are then characterized in terms of the effects of the basic design parameters, $\lambda _s$, $\phi$ and the pore-scale resolution $\lambda _s/L$, on their permeability using the parallel Lattice-Boltzmann (LB) model, presented in a previous study^55,56^. The numerical model recovers the solution of the incompressible Navier-Stokes and continuity equations in the limit of creeping flow (i.e. when $\textrm{Re}=\frac{u_d D_p}{\nu } \rightarrow 0$, where $\nu$ is the kinematic viscosity of the fluid, $u_d$ is the Darcy-scale velocity and $D_p$ is the mean pore-scale diameter), where the Darcy equation is valid. This allows for the calculation of the flow field at sub-pore-scale resolution and the upscaling of the results using volume averaging to calculate the permeability of the domains.

**Figure 3:**
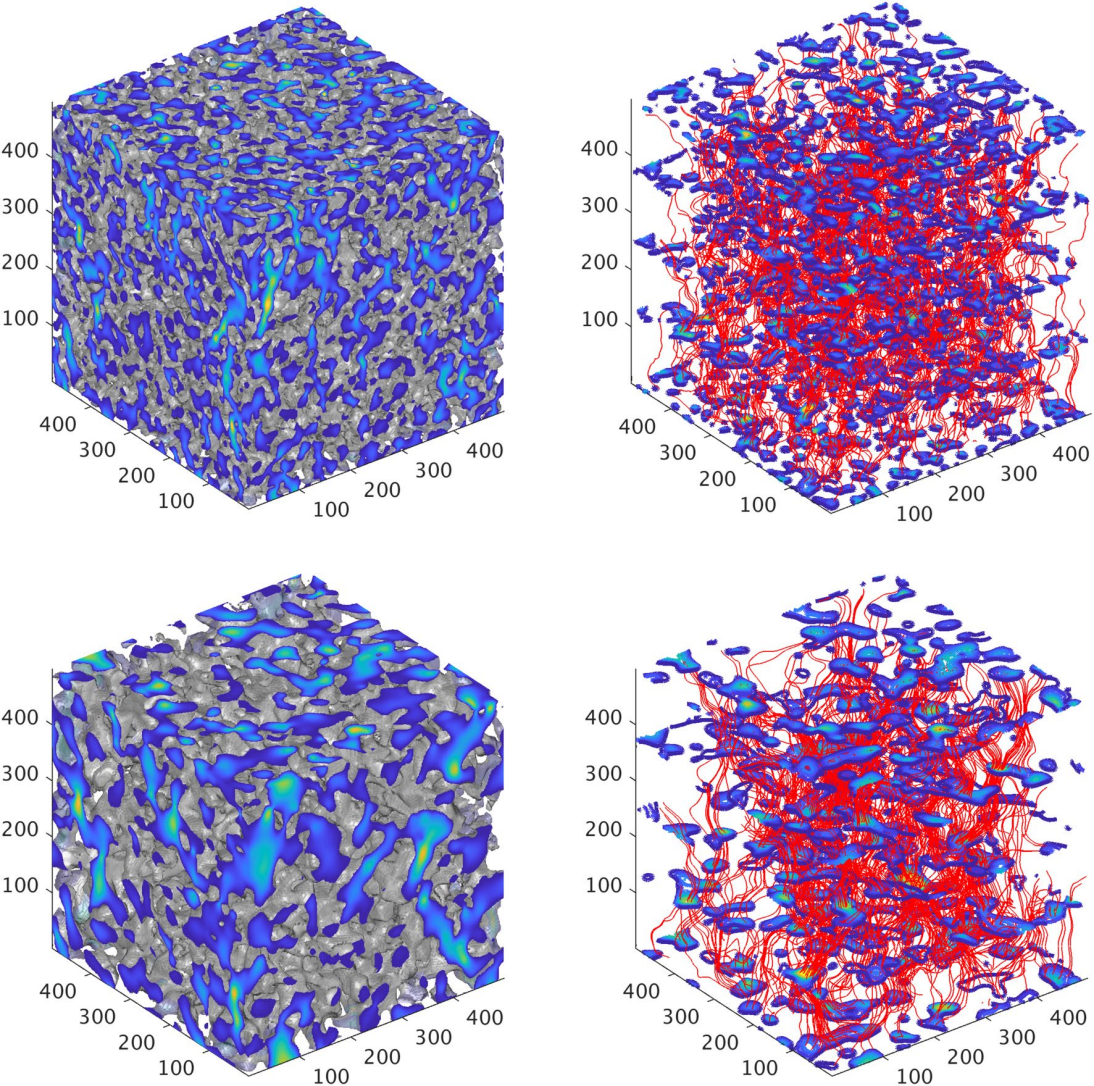
Numerical solution of sub-pore-scale flow in the z-direction using the Lattice-Boltzmann method for two domains of size $L=500\,\delta x$ with different $\lambda _s$ and $\phi =0.45$. (Left) Normalized velocity magnitude through the pores for $\lambda _s=25\,\delta x$, (top) and $\lambda _s=45\,\delta x$ (bottom). Lighter red colors correspond to higher velocities. (Right) Streamlines along the z-direction for $\lambda _s=25\,\delta x$ (top), and $\lambda _s=45\,\delta x$ (bottom). Also shown are the flow contours through the pore at five different layers along the z-direction.

A typical solution of the flow field is shown in Fig. 3 for two original cubic designs of size $L=500\,\delta x$, $\phi =0.45$ and different values of $\lambda _s=25 \,\delta x$ (top), and $\lambda _s=45 \,\delta x$ (bottom). The flow of a fluid with kinematic viscosity equal to $\nu =0.1667\,\delta x^2/\delta t$ is driven by a body force (or an equivalent pressure gradient, $\nabla p$), $\vec {G}=(g_x,g_y,g_z)=\nabla P/\rho$, where $\rho$ is the fluid density. Figure 3(left) show the local velocity magnitude (darker blue colors correspond to lower values, while brighter green colors correspond to higher velocities). Also shown is the solid skeleton in gray color. Figure 3(right) show the corresponding streamlines in red color, as well as the contour surfaces of the velocity magnitude within the pore space in five different layers along the z-direction. These figures clearly reveal that higher velocity magnitudes are observed in the central part of the pores, while the velocity at the solid-fluid surfaces are practically equal to zero. At the same time, a larger number of contour surfaces in the $\lambda _s=25 \,\delta x$ domain [see Fig. 3(top right)] compared to the $\lambda _s=45 \,\delta x$ domain [see Fig. 3(bottom right)] reveals the significantly larger number of pores in the first case. However, given that porosity remains the same between the two, it would be insightful to consider that in reality the $\lambda _s=45 \,\delta x$ domain corresponds to a magnification (i.e. zoom-in) of the $\lambda _s=25 \,\delta x$ domain by a factor of 1.8 (corresponding to the ration of the $\lambda _s$ values). It is also interesting to note that the tortuosity of the streamlines appears to be independent of $\lambda _s$ in these plots. This result is also consistent with $\lambda _s$ representing a magnification parameter of the pore-scale structure, when all other parameters are fixed.

The Darcy-scale (superficial) velocity is then calculated by volume-averaging the sub-pore-scale (interstitial) velocities, $\vec {u}(\vec {x})=(u_x(\vec {x}),u_y(\vec {x}),u_z(\vec {x}))$, as follows;

5 $$\begin{aligned} u_{d,i}=\frac{\int _{V} u_{i} dV}{\int _{V} dV}=\frac{\sum _{x=1}^{N_{x}} \sum _{y=1}^{N_{y}} \sum _{z=1}^{N_{z}} u_{i}(x,y,z) }{N_{x}N_{y}N_{z}} \end{aligned}$$

where $$i=x,y,z$$

Given the calculated Darcy velocity components, $\vec {u}_d=(u_{d,x},u_{d,y},u_{d,z})$, the intrinsic permeability of the medium in all space directions is then derived from Darcy’s law as follows;

6 $$\begin{aligned} K_i=\frac{u_{d,i}\nu }{g_i} \end{aligned}$$

We should note here that the digital domains are isotropic by design (assuming that they are large enough to contain a representative number of pores) and it is therefore expected that they exhibit the same permeability in all three directions.

#### Effects of pore-scale resolution and domain size

**Figure 4:**
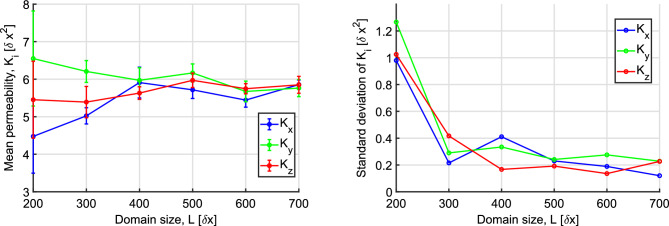
(Left) Effect of the domain size, *L* on the permeability, as calculated by the lattice-Boltzmann method. The curves correspond to the average values of $K_x$, $K_y$ and $K_z$, respectively, for three random domain realizations for fixed $\lambda _s=45\,\delta x$, a domain size in the range of $200\,\delta x\le L \le 700\,\delta x$, and $\phi =0.45$. (Right) The standard deviation of the permeability measurements over all domain realizations vs the domain size in all three flow directions.

The domain size, *L*, and the pore-to-domain-size ratio, $\lambda _s/L$, are both expected to play an important role in the accuracy of our permeability calculations using the previously described numerical model. We thus initially performed a series of simulations on different cubic domain realizations in order to evaluate the sensitivity of our results to these parameters and select an appropriate resolution range for the subsequent numerical study. Given that the number of pores in every domain is expected to be a decreasing function of both the porosity, $\phi$, and the correlation length, $\lambda _s$, we consider a design with $\phi =0.45$ and $\lambda _s = 45\,\delta x$ as the worst case scenario used in this comparison. We then varied systematically both $\lambda _s$ and the domain size *L* towards smaller values. Three different realizations were studied for every set of parameter values and the average permeabilities for these three realizations are reported here. Fig. 4 shows the mean directional permeability ($K_x, K_y$ and $K_z$) for domain sizes ranging between $200\,\delta x \le L \le 700\,\delta x$ with a fixed $\lambda _s=45\,\delta x$. As expected, all permeabilities converge to the same value ($K \approx 6 \,\delta x^2$), as the size of the domain increases and the latter contains more pores. Given that the pore scale resolution is the same in all realizations, we conclude that for values $L \ge 300\,\delta x$ the domains contain a statistically sufficient number of pores and thus the domains correspond to effective REVs that are suitable for our study. Furthermore, the decreasing standard deviation of the measured permeabilities for that range of domain sizes clearly demonstrates the isotropic nature of the domains.

**Figure 5:**
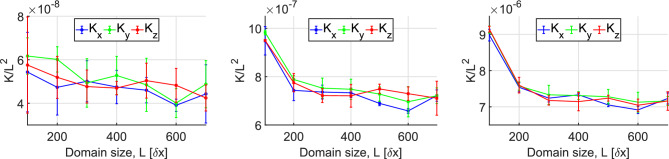
Effects of pore-scale resolution on the normalized permeability $\hat{K} = dx^2/L^2$ results for different values of cubic domain size, $100\,\delta x \le L \le 700\,\delta x$ but a fixed ratio of the $\frac{\lambda _s}{L}=\frac{1}{20}$. The curves correspond to the average permeability values,$K_x$,$K_y$ and $K_z$, respectively, over 3 random domain realizations. (From left to right) $\phi =0.15,0.25$, and 0.45.

Figure 5 shows the effects of the pore-scale resolution on the measured permeability for three different porosity values, i.e. $\phi =0.15, 0.25$ and 0.45, for a fixed value of the pore-to-domain resolution equal to $\frac{\lambda _s}{L}=\frac{1}{20}$. In this way, we keep the number of pores constant over all realizations, while we vary the pore-scale resolution, as $\lambda _s=L/20$. Therefore, $\lambda _s=30\,\delta x$ for $L=600\,\delta x$, while it becomes equal to $\lambda _s=5\,\delta x$ for $L=100 \,\delta x$ for all values of $\phi$ considered here. As previously, the plotted directional permeabilities are averaged over three different domain realizations and their values are normalized with the domain size $L^2$.

Here, we also observe the important role of the domain porosity on the minimum domain size required to obtain a convergence of the directional permeability curves to a size-independent value. It appears that the permeability curves converge faster for lower values of $\phi$ (i.e. by comparing the results for $\phi =0.25$ with those for $\phi =0.45$). We argue that this result is due to the larger number of pores that are contained in the domain, as $\phi$ decreases (for fixed *L*), thus rendering the domain more representative even for small sizes. For the case, however, of $\phi =0.15$, it appears that the calculated permeabilities exhibit a significant standard deviation over the entire range of domain sizes, despite the fact that the number of pores should be much higher than those for the other two values of $\phi$. We postulate that this is due to the increased sensitivity of the calculated permeability when the porosity approaches a critical value, $\phi _c$, where the domain is no longer percolating in all three direction. We also address this issue later in this article and provide an estimate for the value of $\phi _c$. Based on the previous analysis, we conclude that we obtain representative permeability values for domain sizes $L \ge 300 \,\delta x$.

#### An expression for the permeability of the cubic domains

Before proceeding to the construction and characterization of the actual 3D micromodels, it is useful to derive an expression for the Darcy-scale permeability of the periodic cubic domains as a function of the two main design parameters, i.e. the porosity, $\phi$, and the correlation length, $\lambda _s$.

Based on the analysis in the previous sections, we have concluded that the LB converges to fixed permeability values for $L \ge 300\,\delta x$ and $\frac{\lambda _s}{L}\approx \frac{1}{20}$ (i.e. $\lambda _s\ge 15\,\delta x)$. Under these conditions, the domains correspond to REVs, while the pore scale resolution is sufficiently high in order to produce numerically accurate solutions. We therefore perform a series of LB simulations in the digital cubic domains over a wider range of $\lambda$ and $\phi$ values (a single domain realization for every set of parameter values) for a sufficiently large domain of size $L=500\,\delta x$.

**Figure 6:**
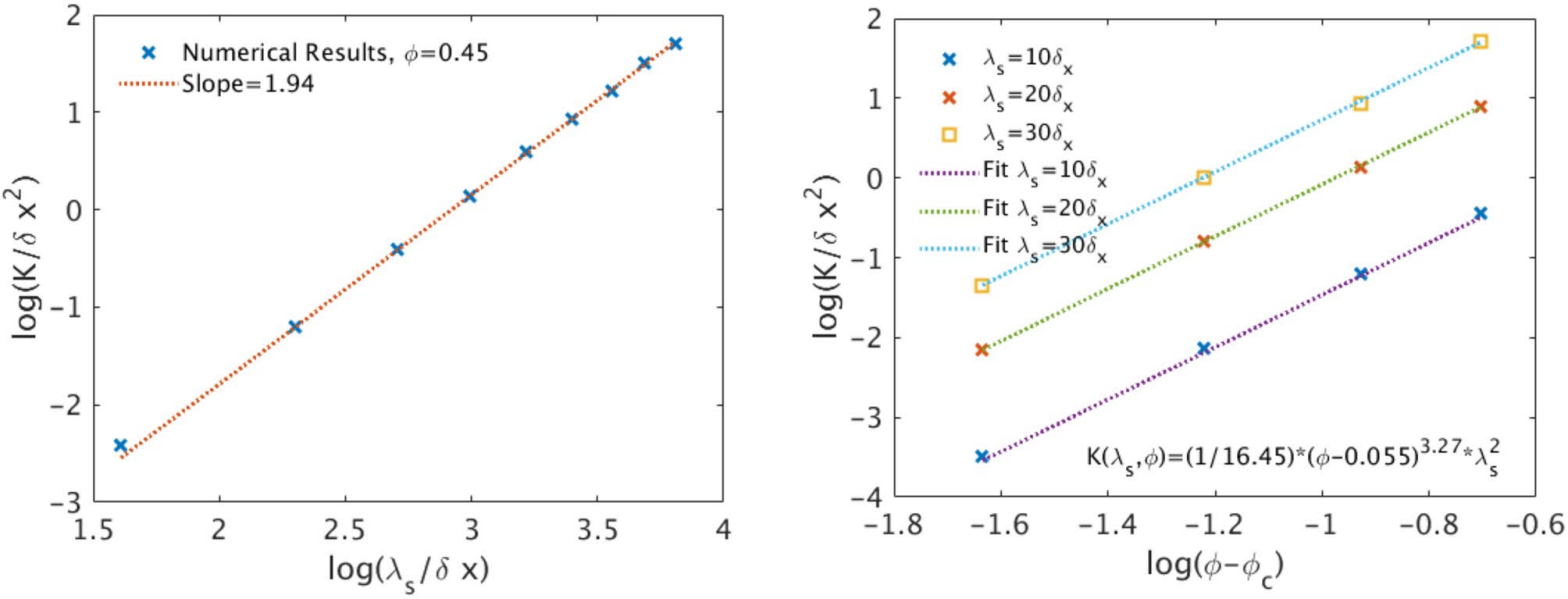
(Left) Average domain permeability $K_{LB}=\frac{K_x+K_y+K_z}{3}$ vs the correlation length $\lambda _s$ for a single domain realization with $L=500 \,\delta x$ and $\phi =0.45$. The dashed curve corresponds to the least square fit over all the data and reveals the scaling $K_{LB}(\lambda _s)\propto \lambda _s^{1.94}$. (Right) Average domain permeability $K_{LB}=\frac{K_x+K_y+K_z}{3}$ vs the effective porosity $\phi -\phi _c$ for three different values of the correlation length $\lambda _s=10, 20$ and $30\,\delta x$ for a single domain realization with $L=500 \,\delta x$. The dotted lines correspond to the least square fit that best describes all data. The corresponding equations is $K_{LB}(\lambda _s,\phi )=\frac{1}{16.45}(\phi -\phi _c)^{3.27} \lambda _s^2$, where $\phi _c=0.055$.

Figure 6(left) shows the dependence of the numerically measured permeability as a function of $\lambda _s$ in a logarithmic plot for a fixed porosity value equal to $\phi =0.45$. Given the isotropic nature of the domain, the permeability here is averaged over all three directions, as $K_{LB}=\frac{K_x+K_y+K_z}{3}$. Figure 6(left) shows that the permeability follows a power-law scaling with $\lambda _s$, as $K_{LB}\propto \lambda _s^{1.94}$. This result is in excellent agreement with the expected scaling exponent of 2, given that the permeability should scale with the second power of a characteristic length scale (here $\lambda _s$).

The effect of porosity on the permeability is also implemented by first considering that the domain should become impermeable, when the porosity falls below a critical value, $\phi _c$^57,58^. Figure 6(right) shows the permeability vs $\phi -\phi _c$ for $\phi _c=0.055$ for three different values of $\lambda _s$. A least square fit over all simulations shows that the dependence of the permeability on both design parameters is very well described by the following expression;

7 $$\begin{aligned} K_{LB}=a (\phi -\phi _c)^\beta \lambda _s^2 \end{aligned}$$

where $\alpha =\frac{1}{16.45}$ and $\beta =3.27$. It is interesting to note that the value of the scaling exponent $\alpha$ is in very good agreement with the value of 3.05 experimentally determined by Bourdie et al.^59^ for Fontainebleau sandstone and the value of 3.25 reported by Zinszner^57^ for “normal” sandstone. We thus conclude that the previously described stochastic reconstruction algorithm reproduces very accurately the permeability of sandstone (as measured numerically using the Lattice-Boltzmann method), at least for the specific range of parameter values. In the following sections, any reference to numerical LB results is calculated based on the fitting expression of Eq. (7), unless otherwise stated.

#### Comparison with the Kozeny–Carman formulation

It is also interesting to correlate our numerical results with the predictions of the Kozeny–Carman formulation;

8 $$\begin{aligned} K_{KC}=\frac{\phi ^3}{k_0 \tau _h^2 S_p^2} \end{aligned}$$

where $S_p=A_w/V_t$ is the specific surface of the porous domain, defined as the wetted surface, $A_w$, per unit volume, $V_t$, of the medium^60^, $\tau _h$ is the hydraulic tortuosity, defined as $\tau _h=L_e/L$^61^, and $k_0$ is a prefactor characteristic of the pore-scale structure. $L_e$ denotes the actual average length of the streamlines in the dominant flow direction, while *L* denotes the distance between any two points along the same streamlines. The above formulation was derived by Kozeny and Carman^62,63^ using an equivalent hydraulic diameter, $D_h$, approach in Darcy’s law that led to the expression;

9 $$\begin{aligned} K_{KC}=\frac{\phi D^2_h}{16k_0\tau ^2_h} \end{aligned}$$

Given that the hydraulic diameter is by definition $D_h=4V_v/A_w=4\phi V_t/A_w=4\phi /S_p$, where $V_v$ is the void volume of the porous domain, Eq. (8) is then derived by replacing the above definition $D_h=4\phi /S_p$ into Eq. (9).

The specific surface is also often defined per unit volume of the solid, $S_o=A_w/V_s$, where $A_w$ is the wetted solid surface and $V_s$ is the corresponding solid volume. It is thus straightforward to show that $S_o=S_p/(1-\phi )$ ($S_o=A_w/V_s =\frac{A_w V_t}{V_s V_t}= S_p/(1-\phi )\rightarrow S_p=S_o(1-\phi )$) and thus the following Kozeny-Carman formulation is equally valid;

10 $$\begin{aligned} K_{KC}=\frac{\phi ^3}{k_0 \tau _h^2 S_o^2 (1-\phi )^2} \end{aligned}$$

The specific surface is explicitly determined in our digital cubic domains by applying the Matlab clustering function regionprops3 to calculate the wetted surface, $A_w^{np}$ (i.e. the void-solid interface of each domain). Given that this clustering algorithm does not account for the periodicity of the solid matrix, but rather includes also the external surfaces in the calculation of $A_w^{np}$, the actual specific surface corresponding to a periodic domain is calculated by the following formula;

11 $$\begin{aligned} A_w=A_w^{np}-6L^2(1-\phi ) \end{aligned}$$

The above formula corrects the calculated value by subtracting the surface area of the solid at the faces of the cubic domain, assuming that they each face is composed by $\phi L^2$ porous surface and $(1-\phi )L^2$ solid surface.

The hydraulic tortuosity, $\tau _h$, is then calculated from the discrete solution of the LB method for the interstitial (i.e. pore-scale) velocity field, $\vec {u}=(u_x,u_y,u_z)$, using the approach proposed by Matyka et al.^61,64^:

12 $$\begin{aligned} \tau _h=\frac{\langle |\vec {u}|\rangle }{\langle |\vec {u} \cdot \vec {e_z}| \rangle } \end{aligned}$$

where $\langle |\vec {u}|\rangle$ is the average interstitial velocity magnitude and $\vec {e_z}$ is the unit vector in the dominant flow direction. Therefore, the denominator stands for the average interstitial velocity along the dominant flow direction.

**Figure 7:**
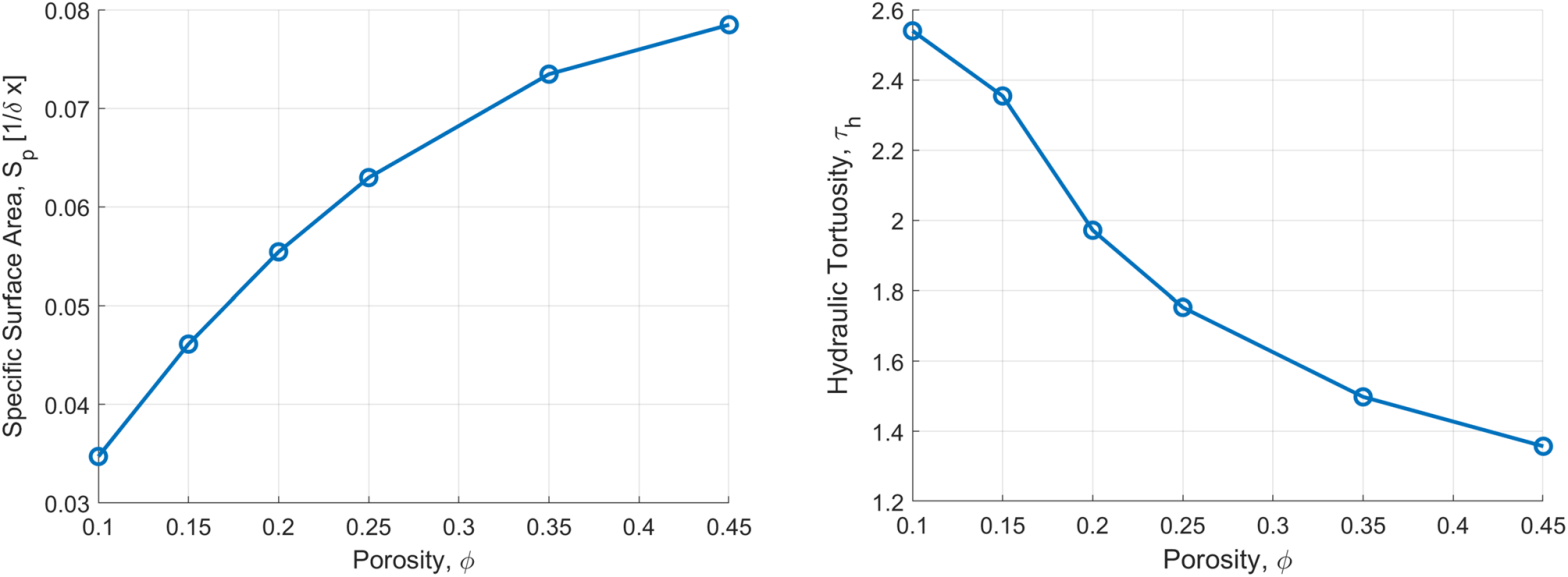
Parameters for the Kozeny–Carman equation (Eq. 10) calculated over three different domain realizations for $L=500 \,\delta x$ and $\lambda _s=25 \,\delta x$. (Left) Average specific surface (per unit solid), $S_o$, as a function of the domain porosity, $\phi$. (Right) Average hydraulic tortuosity for the same domain as calculated by applying Eq. (12) on the pore-scale velocity field solution using the LB method.

**Figure 8:**
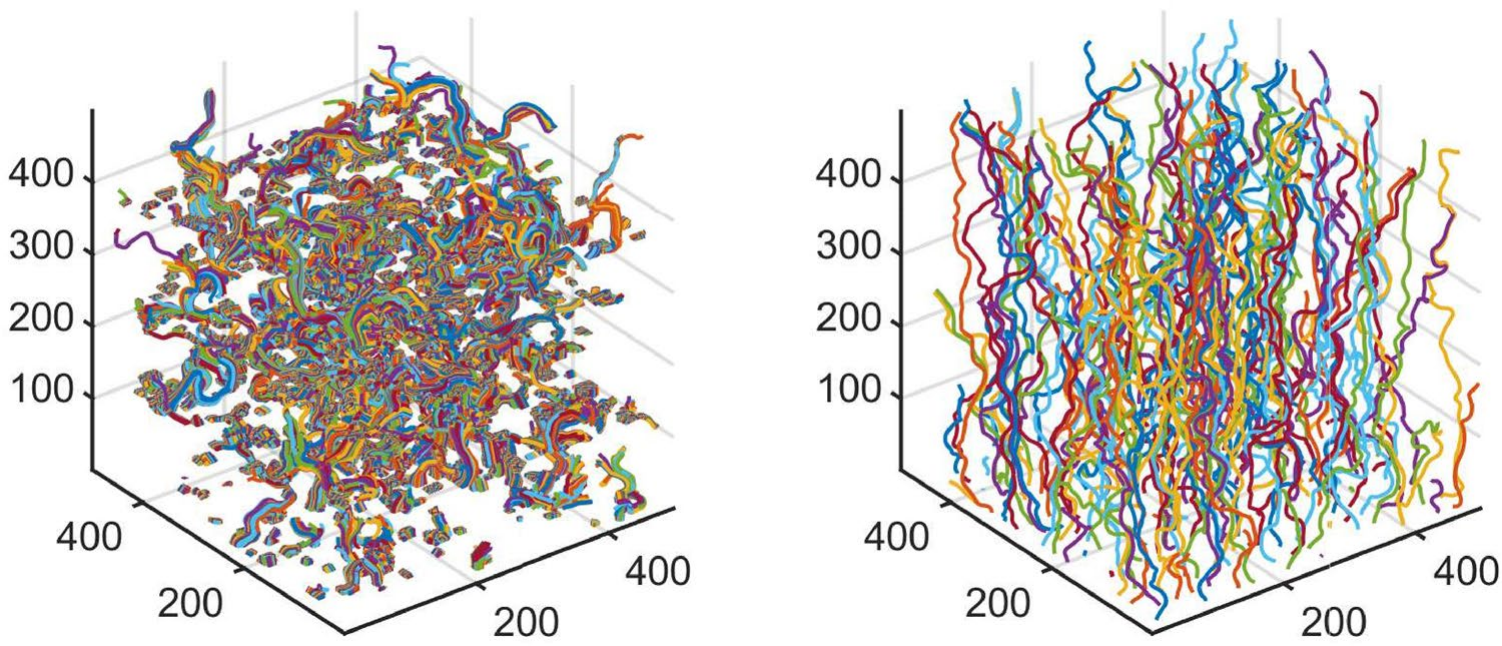
Flow streamlines for two domains of size $L=500\,\delta x$ and $\lambda _s=25 \,\delta x$ but different porosities, $\phi =0.15$ (left), and $\phi =0.45$ (right). The principal flow direction is along the z-axis.

Figure 7 shows the the specific surface, $S_p$, and hydraulic tortuosity, $\tau _h$, as a function of porosity $\phi$ for $\lambda _s=25\,\delta x$, as calculated using this approach. In Fig. 7(right) we observe that the hydraulic tortuosity becomes higher as $\phi$ decreases. The tortuosity is found equal to $\tau =2.4$ for $\phi =0.15$, while it decreases significantly to a value of $\tau =1.4$ for $\phi =0.45$, corresponding to less tortuous streamlines. This is very clearly demonstrated in Fig. 8 that shows the flow streamlines for these two values of $\phi$.

**Figure 9:**
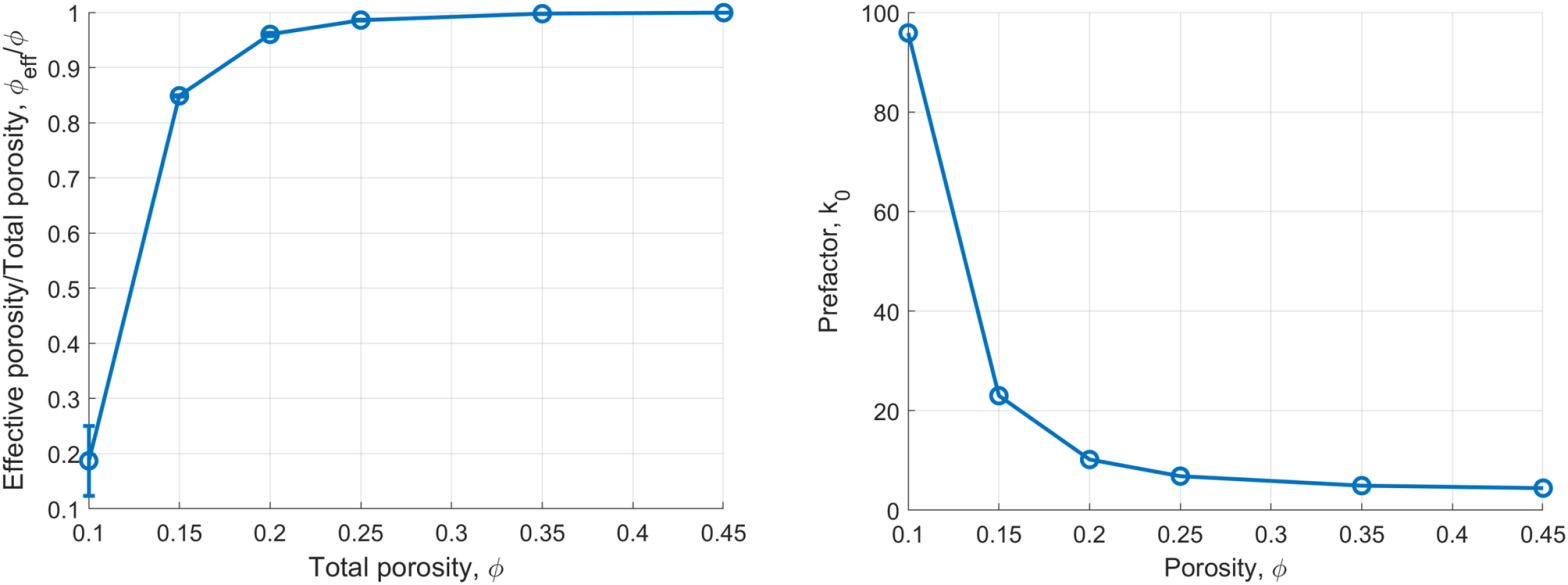
(Left) Ratio of the percolating (effective), $\phi _{eff}$, over the total porosity, $\phi$. (Right) Mean value of the parameter, $k_0$, of the Kozeny-Carman formulation (Eq. 10) that recovers the same value of permeability, as the one calculated using the LB method.

Figure 9(left) shows the percolating fraction of the pore space measured by counting the connected pores at the top and bottom surfaces, as well as those connected to any boundaries of the cube. The plot reveals that for $\phi \ge 0.15$ practically all pores are interconnected to a single percolating void cluster. For smaller values of $\phi$, however, the fraction of the void space that belongs to the percolating cluster decreases very sharply, reaching a value of $\frac{\phi _{eff}}{\phi } \approx 0.2$ for $\phi =0.1$. This result further supports the existence of a critical porosity value, as also discussed previously, where the domain becomes practically impermeable.

**Figure 10:**
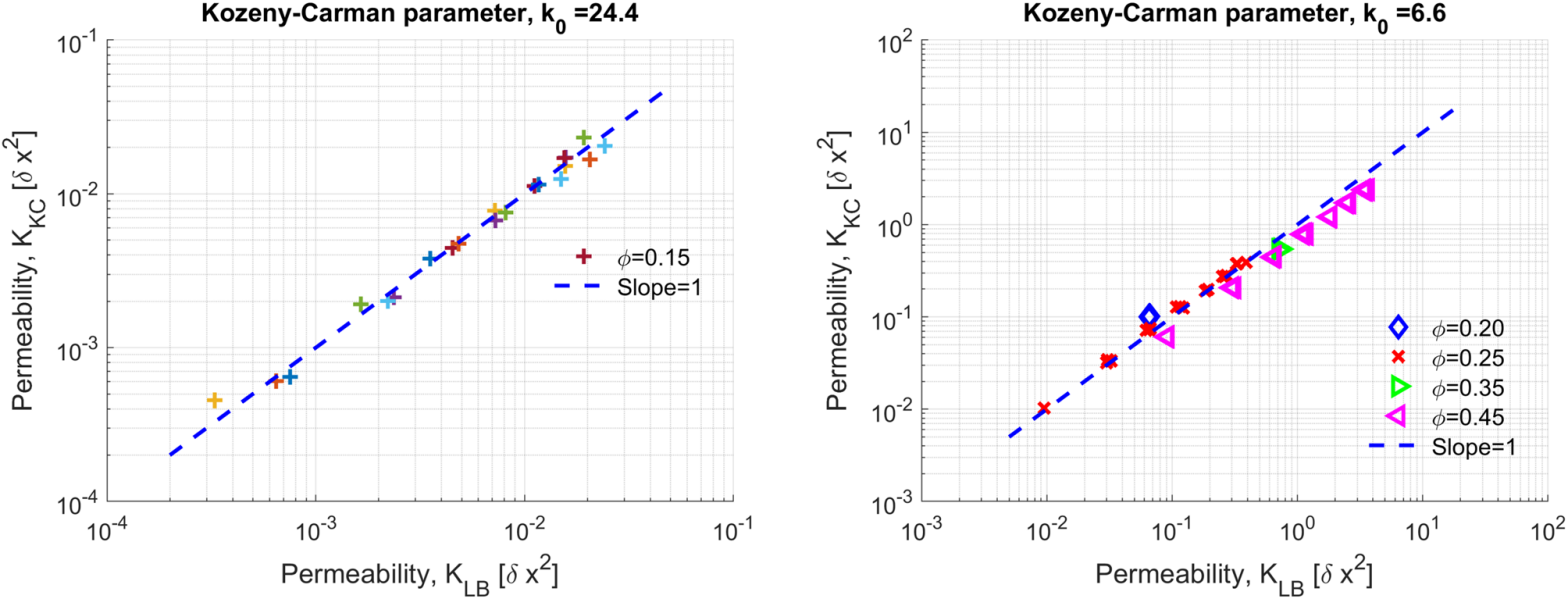
(Left) Comparison between the calculated permeability by the Kozeny–Carman equation for $k_0=33.7$ for all the simulations at $\phi =0.15$ and different values of $\lambda _s$ and *L* with the results of the Lattice-Boltzmann simulations for the same domains. (Right) Comparison between the calculated permeability by the Kozeny–Carman equation for $k_0=13.6$ for all the simulations where $0.20\le \phi \le 0.45$ and different values of $\lambda _s$ and *L* with the results of the lattice-Boltzmann simulations. The dashed curve in both plots corresponds to equal permeability values.

Figure 9(right) shows the value of the prefactor $k_0$ for the Kozeny–Carman equation that gives the best match with the numerically calculated values of the permeability of the LB method. Interestingly enough, the calculated prefactor appear to be a function of $\phi$ for values $\phi \le 0.20$. For larger values, the effect of $\phi$ is practically negligible. Based on this result, we determine a universal value for the prefactor $k_0=6.6$ in Eq. (10) by fitting with the values of the permeability determined by Eq. (7). Figure 10(right) shows that practically all our results for $\phi \ge 0.20$ (and regardless of $\lambda _s$ and *L*) can be well described by the Kozeny–Carman equation for the same prefactor value of $k_0=6.6$.

Figure 10(left) shows the very good match between the numerical LB results and the Kozeny–Carman equation for $\phi =0.15$ (regardless of $\lambda$ and *L*) when a prefactor equal to $k_0=24.4$ is used in Eq. (10). We therefore postulate that there is a strong dependence of $k_0$ on $\phi$ for values close to the critical porosity value, where the effective (i.e. percolating) fraction of the porosity decreases sharply with respect to the total one. When $\phi>> \phi _c$, the prefactor becomes progressively a weaker function of $\phi$ and is practically constant over a wide range of porosity values.

#### Pore-scale velocity correlations in periodic cubic domains

As a last step of our numerical study, we study the effects of $\lambda _s$ on the pore-scale correlation of the velocities, as calculated using the LB method. For this purpose, we utilize the variogram function $\gamma (\mathbf{{r}})$ that commonly used to estimate the spatial correlation of a variable (the mean velocity here) in geostatistical studies. This approach provides equivalent information with the covariance function. The variogram is more straightforward to calculate numerically than the covariance function as it pertains to the value differences between pairs ^65^.If *X* is a random field, the *empirical variogram* is usually derived from Eq. (13)

13 $$\begin{aligned} \gamma (\mathbf{{r}}) = \frac{1}{2} E \left[ \lbrace X^{\prime }(\mathbf{{s}})- X^{\prime }(\mathbf{{s+r}})\rbrace ^2\right] , \end{aligned}$$

where *E* is the expected value (the mean value) and $(\textbf{s}, \textbf{s}+r)$ are all data pairs with a distance $\textbf{r}$.

A theoretical *variogram model* is fitted on the empirical variogram to account for spatial continuity. This theoretical variogram model is defined by a small number of parameters (sill, nugget effect and range or correlation length) and can give an approximation of the variogram value for all possible distances. In our case, we used Matlab’s optimization toolbox to estimate the best-fitting parameters of the Gaussian variogram of Eq. (14),

14 $$\begin{aligned} \gamma (\mathbf{{r}})=\sigma ^2 \left( 1-e^{-3 |r|^2 / \lambda ^2} \right) +c_0, \end{aligned}$$

where $\sigma ^2$ is the variance, $c_0$ is the nugget effect and $\lambda$ is the correlation length or variogram range. The correlation length is the maximum distance of spatial dependence.

**Figure 11:**
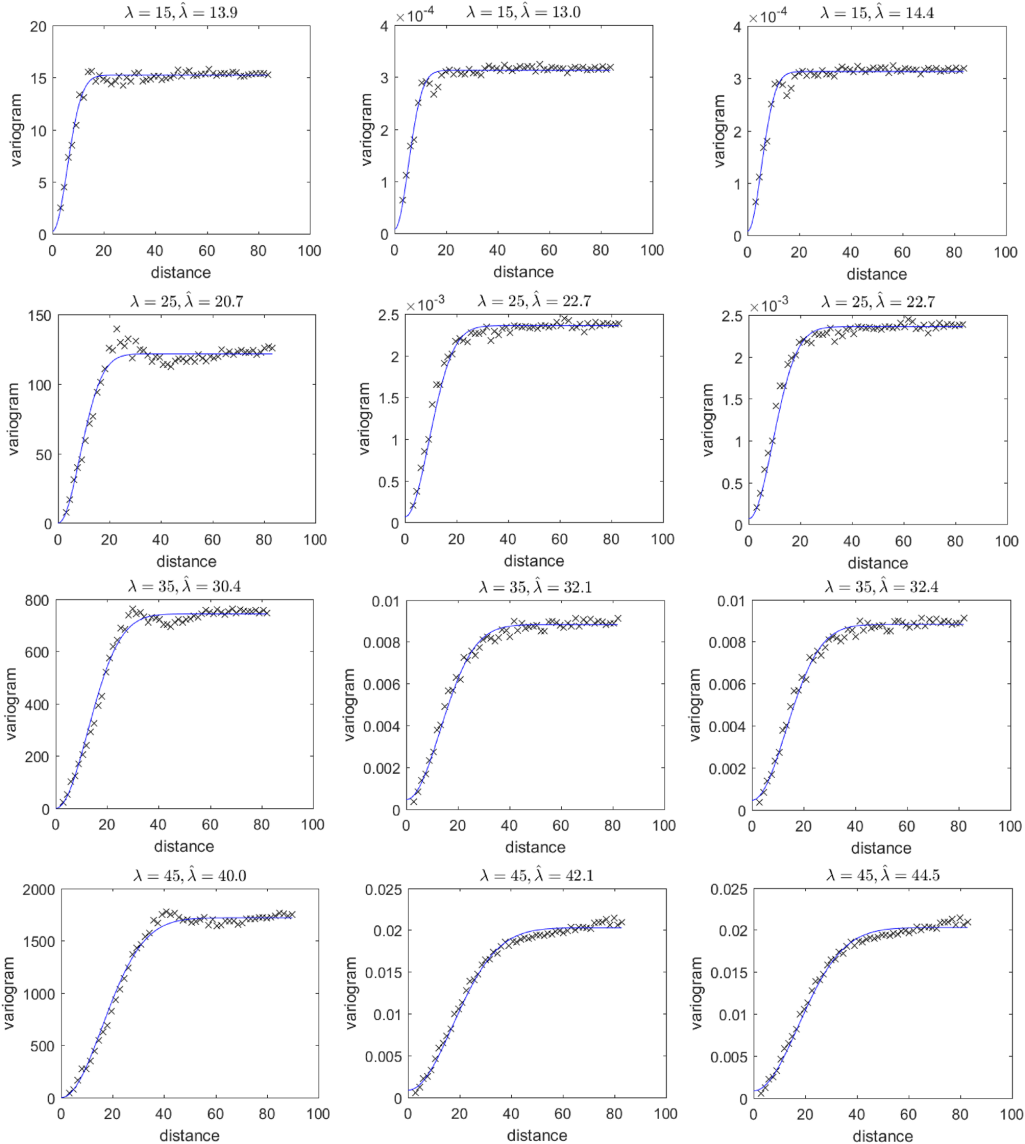
3D variograms of the velocity magnitude (as calculated using the LB model) for different values of $\lambda =\lambda _s$ and porosity $\phi$. The domain size is $L=500\,\delta _x$ in all cases and the calculated correlation length is reported over each plot as $\hat{\lambda }$ alongside the corresponding $\lambda _s$ value of each domain. (Left column) $\phi =0.15$, (middle column) $\phi =0.25$, and (right column) $\phi = 0.35$.

In this work, the empirical variogram was estimated from a random sampling of 25,000 points of the simulated velocity magnitude in cubes of $L=500\, \delta x$. For each porosity value ($\phi = 0.15, 0.25$ and 0.35) there are four different velocity magnitude fields, each with a different $\lambda$ value ($\lambda =15, 25, 35$ and $45\,\delta x$). Estimating the parameters of the model that gives the best fit to the empirical variogram, it is possible to estimate the correlation length $\hat{\lambda }$ for each such field in order to validate the results of the simulation. The variograms and the estimated correlation lengths, $\hat{\lambda }$, are given in Fig. 11.

The variograms and the estimated correlation lengths, $\hat{\lambda }$, are given in Fig. 11. Our results show that the correlation lengths derived by the variograms match very well the design parameter, $\lambda _s$, that was used for each domain realization, regardless of the value of the domain porosity, $\phi$. These results show that the magnitude of the pore-scale velocity is strongly correlated for distances less than the correlation length $\lambda _s$, as expected. Over longer distances, however, the calculated velocities are clearly uncorrelated.

## Additive manufacturing and X-ray imaging of the 3D micromodels

### 3D printing methodology

**Figure 12:**
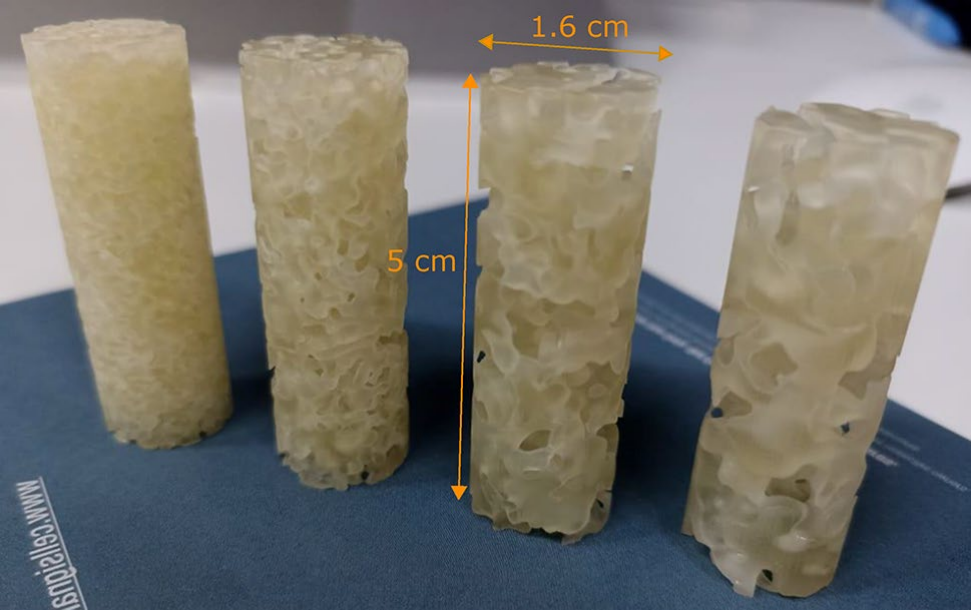
Snapshot of the 3D printed cylindrical porous micromodels, according to the designs of Fig. 2. The actual height, *L* of the micromodels ranges from 4.9 to 5 cm, while the diameter, $D_0$, is practically equal to 1.6 cm, in all cases. (From left to right) $\lambda _s=15\,\delta x$, $\lambda _s=25\,\delta x$, $\lambda _s=35\,\delta x$, and $\lambda _s=45\,\delta x$.

The cylindrical 3D micromodels (designed as discussed in Section Stochastic reconstruction of digital porous domains) were fabricated using a commercially available ProJet MJP 3600 3D printer by 3D SYSTEMS that utilizes the material jetting (MJ) technology. This approach relies on spraying hundreds of tiny droplets of a photosensitive polymer resin at the same time, following the digital design of the porous domain. The droplets are subsequently solidified using multiple UV light sources, while a dissolvable wax is also sprayed in the void spaces as a support material for the actual printed parts. The photopolymer resist used for the fabrication of the micromodels was VisiJet M3, while the support material was VisiJet S300. The micromodels were constructed with a layer thickness of $16\,\upmu \hbox {m}$ (in the vertical axis) and a typical resolution of $25\,\upmu \hbox {m}$ along the horizontal directions. After the printing was completed, a post-processing step was followed that included placing each micromodel in a vacuum oven at 55^∘^ C for 2 hours in order to remove the supporting wax.

Figure 12 shows a snapshot of the printed 3D micromodels, according to the digital designs shown in Fig. 2. with correlation lengths from left to right $\lambda _s=15\,\delta x$, $\lambda _s=25\,\delta x$, $\lambda _s=35\,\delta x$ and $\lambda _s=45\,\delta x$. The actual height *L* of the micromodels ranges from 4.9 to 5 cm while their diameter $D_0$ is 1.6cm in all cases.

### X-ray imaging of 3D micromodels

**Figure 13:**
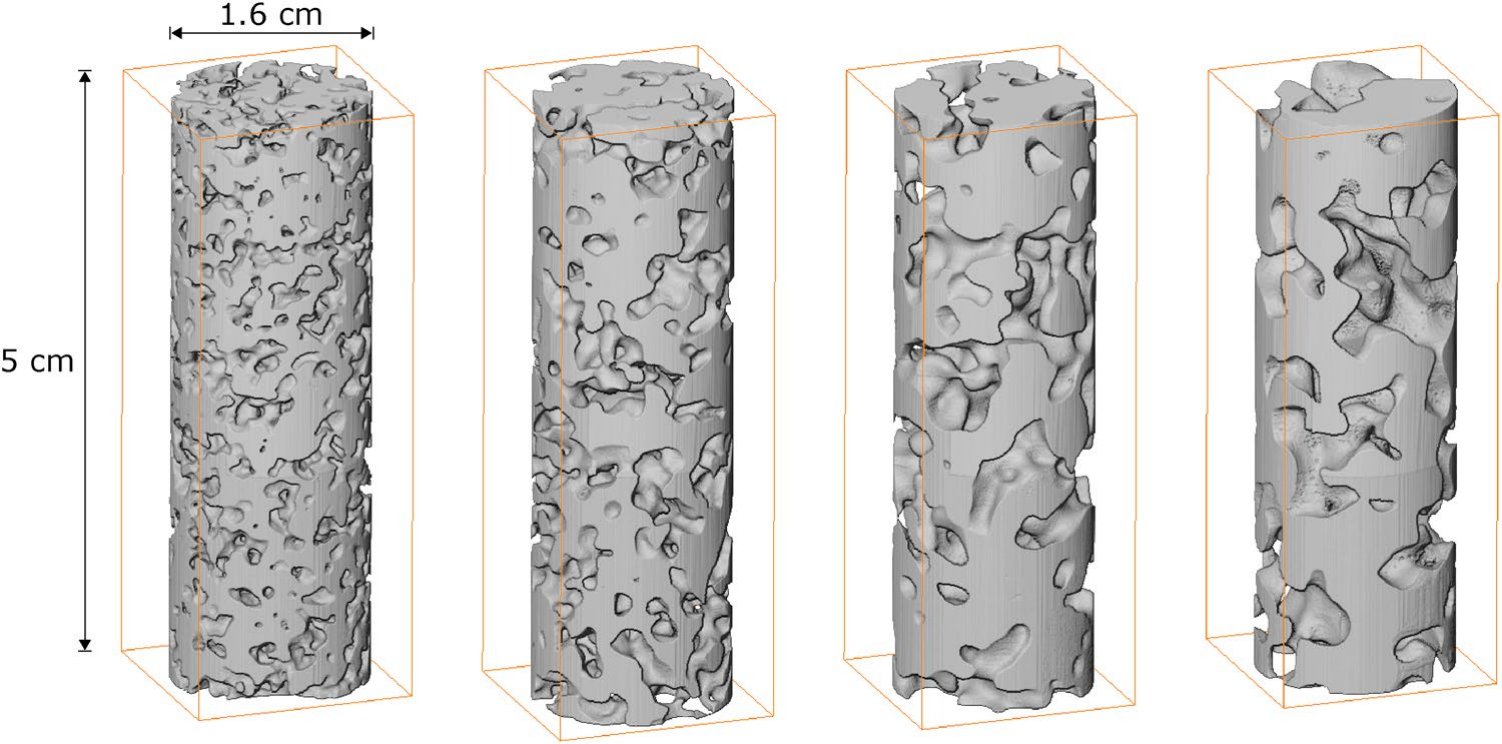
Snapshot of 3D scanned samples. (from left to right) $\lambda _s=15\delta x$, $\lambda _s=25\delta x$, $\lambda _s=35\delta x$, and $\lambda _s=45\delta x$.

The 3D micromodels were then scanned using the open and modular micro-XRCT-system^66^. All samples were scanned with identical settings: X-ray tube voltage 90 kV, X-ray tube flux 90 kV, detector exposure time 1500 ms (Shad-o-Box 6K HS detector employed), and 1800 equidistant projection angles with 2 slightly in-plane shifted detector positions for bad detector pixel compensation, cf.^66^. The geometric magnification was set to 3.54 corresponding to a uniform voxel edge length of ${14}\,\upmu \hbox {m}$. The resulting field of view was ${41.16 \times 41.16 \times 32.256}\,\hbox {mm}$ (width $\times$ depth $\times$ height) and sufficient to scan the sample over the entire diameter (16 mm), however, too small to scan the samples over the complete length (50 mm). Therefore, first the bottom part and then the top part with a small overlap were scanned. Merging both data sets into one was performed within the binarization step of the reconstructed data sets. The 3D volume reconstructions were done with the Feldkamp-Davis-Kress (FDK) algorithm^67^ implemented into the software Octopus Reconstruction (Version 8.9.4-64 bit)^68^. In this step, an additional software-based ring artifact improvement technique was applied.

An image segmentation was then performed in order to classify the solid-/pore space based on the 3D scans. The Otsu method was adopted for this task utilizing the intensity histogram of the image^69^. Utilizing this method, we obtained a threshold value that maximizes inter-class variance for the various features present in the acquired image. The intensities bigger than the threshold were assigned to solid and the rest were assigned to pores. Misclassified pixels encountered during this process, primarily attributed to the inherent image-related noise of XRCT, were subsequently addressed by eliminating isolated pixels as determined by connectivity. Subsequently, corresponding to the cylindrical shape of the sample, the exterior region of the sample was trimmed. Therefore, only the region of interest of the sample would be considered in the final output of the segmentation. This work was conducted with the Image Processing Toolbox from Matlab^©^^70^ (The MathWorks, Inc.).

Figure 13 shows a 3D reconstruction of the scanned micromodels in the same order with respect to $\lambda _s$ (from left to right), as shown in Fig. 12. The characteristics and the quality of the printed domains with respect to the corresponding digital designs (Fig. 2) are discussed in detail in the “Results and discussion” section.

## Experimental permeability measurements

**Figure 14:**
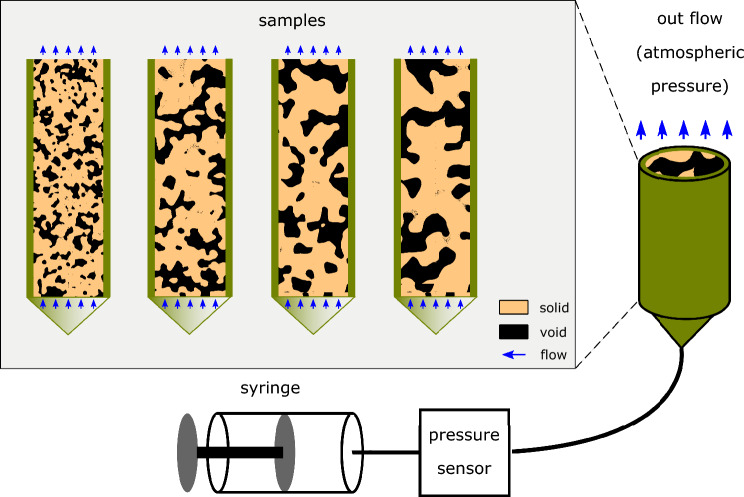
Schematic of the microfluidic setup used in this study to measure the permeability of the 3D micromodels.

The intrinsic permeability of each micromodel was measured experimentally using the microfluidic setup shown in Fig. 14. A solution of 99.5% glycerol with a viscosity of $\mu =1.4 \mathrm {Pa \, s}$ at room temperature was injected from the bottom side of the sample at a constant flow rate, while the top side was kept open to the atmosphere. For that purpose, a setup was prepared bearing a CETONI^©^ neMESYS 1000N syringe pump, in combination with a 1/16” tube to introduce the corresponding fluid into the sample for the measurement. An Elveflow^©^ MPS2, pressure sensor was used at the inlet (bottom) of the sample, with a pressure measurement range from 0 to 1 bar. The sample itself was mounted on a conical adapter, in order to make sure that the sample would come in contact with the working fluid at the same time all over its cross-section. A 1/16” tube fitting was attached to the conical adapter for the introduction of the working fluid, and a shrink tube was fitted externally to the ensemble, with only the 1/16” fitting being exposed. In this way it was made sure that no leakage of the working fluid would take place from the sample.

Given the size of the pores in the samples, and the overall available cross-sectional area independently of the correlation length of the sample, the size of the tube would induce a higher pressure drop than that of the sample itself. Obviously, the effect of the tubing would become increasingly more significant for increasing correlation lengths. To overcome this challenge, the measurement of the intrinsic permeability of each sample would take place in two steps. In the first step, paired values between the applied fluxes and the induced pressure drops were taken from the setup, which was at the state as described above. As soon as the limits of the sensor in terms of measured pressures was reached, the measurements would stop. Then, in the second step, the shrink tube holding the sample would be cut-off and removed with the sample. Then, the same fluxes as before were applied, while the induced pressures were measured with the sample being absent. In this way, we were able to strip the sample pressure measurements from the background of the fluid introduction setup, by subtracting the pressure measurements acquired in the second step from the those acquired in the first step, for the same applied fluxes.

The results of the experimental measurements are shown in Table 3 and are discussed in detail in the following section.

## Results and discussion

The main objective of our study was to design, construct and validate 3D porous media micromodels with fine-tuned pore-scale characteristics, that are representative of actual REV sandstone samples. The effects of the pore-scale parameters, including the correlation length and the porosity, on the REV-scale permeability of the domains were studied using a LB flow simulator. By solving the flow problem at sub-pore-scale resolution in the limit of creeping flow, we calculated the intrinsic permeability as function of $\phi$ and $\lambda _s$. Our numerical LB study combined with the velocity magnitude variograms has demonstrated that the digital domains represent REVs and recover the solution of Kozeny-Carman formulation, as long as the digital designs contain a sufficiently large number of pores. Given the current technological limitations in the spatial resolution of state-of-the-art additive approaches, this condition is met when the digital domain size is $L \ge 300$, while the pore-scale resolution lies in the order of $\frac{\lambda _s}{L}=\frac{1}{20}$.

Given that the cylindrical domains were extracted from much larger representative cubic samples, we verified numerically a potential deviation of the cylindrical designs from their respective cubic ’parents’. This was achieved by calculating the permeability of both the cubic and the resulting cylindrical designs using the LB method, as previously discussed. Table 2 shows the numerically measured permeability and porosity of the cubic and the extracted cylindrical domains along the z-direction. While the porosity of both designs is practically the same (equal to $\phi \approx 0.45$), the permeability of the cylindrical domains is found to differ from that of the cubic ones (taking typically lower values), especially for larger values of $\lambda _s$. This should be attributed to the finite size effects along the perimeter of the cylindrical domains, that become more pronounced as the pore size becomes comparable with the diameter of the cylinder. This effect should be taken into account during the design of such micromodels.

The representativeness of the printed domains compared to the digital designs is very well demonstrated by a visual comparison of (a) the cylindrical designs shown in Fig. 2, (b) the shapshots of the printed micromodels shown in Fig. 12, and (c) the 3D reconstruction of the printed domains using X-ray imaging (as shown in Fig. 13. These figures reveal an excellent match between the original designs and the physical 3D micromodels (at least macroscopically) with an evident effect of the selected value of the correlation length $\lambda _s$ on the size of the apparent pore bodies at the perimeter of the micromodels.

**Figure 15:**
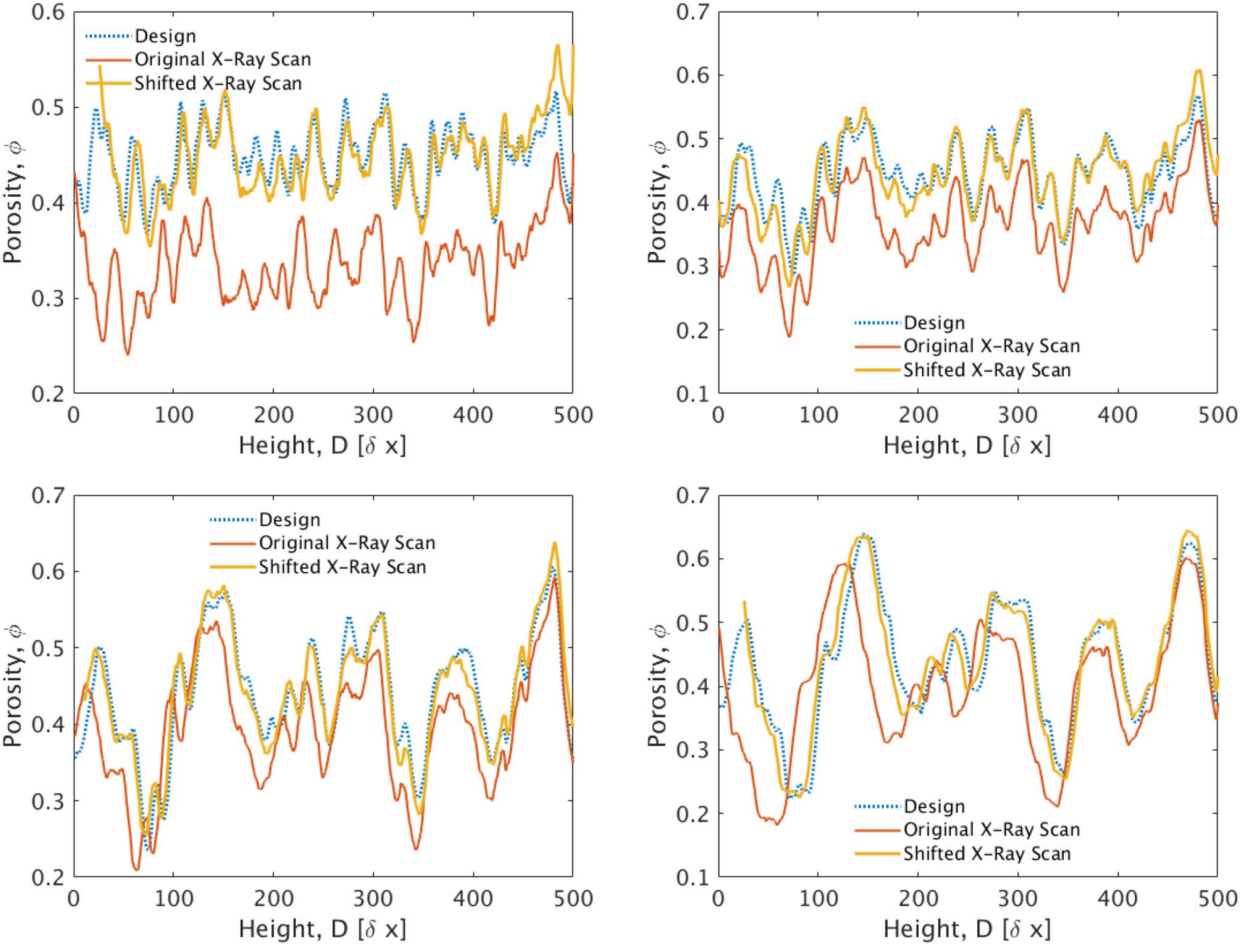
Comparison of the x–y averaged porosity over the height, *L* (z-direction) between the X-ray scanned micromodels and the cylindrical designs. The continuous red curve corresponds to the X-ray measurement, the dotted blue curve corresponds to the original design, while the continuous orange curves results after shifting the X-ray curve by $\phi _{Xray}+\Delta \bar{\phi }=\alpha (1-z/L)$, where $\alpha$ is a prefactore representing the compression of the printed micromodel compared to the original, and $\Delta \bar{\phi }$ is the different between the design and actual porosity of the micromodel, as recovered using XRCT. (Top left) $\lambda _s=15\,\delta x$, (top right) $\lambda _s=25\,\delta x$, (bottom left) $\lambda _s=35\,\delta x$, and (bottom right) $\lambda _s=45\,\delta x$.

A more in-depth study of the X-ray imaging results allows for assessing the quality of the internal structure of the printed domains with respect to their designs. Fig. 15 shows the mean (x–y averaged) porosity of the four micromodels as a function of the distance, *D*, from the top side (along the z-direction). Here, the dotted blue curves correspond to the digital design, while the continuous red curves correspond to the results obtained using X-ray imaging. The main conclusion from this comparison is that the porosity profiles of the printed samples match quite well those of the digital designs, although they appear to be shifted towards an overall lower mean value. The divergence from the input porosity is found to be more pronounced at lower values of $\lambda _s$. The mean porosity of the printed samples, as measured using X-ray imaging, is shown in Table 3. Here we observed a divergence of almost 10% for $\lambda _s=35\delta x$ and $45\delta x$, while the divergence for $\lambda _s=15\,\delta x$ is approximately 26%. By shifting the X-ray porosity curves by the difference, $\Delta \bar{\phi }(\lambda _s)$ between the digital domain porosity and the one of the printed sample, we obtain the continuous orange curves of Fig. 15 that match very well with the designs. We should also note that a re-scaling is also applied along the depth axis of this plots to accommodate for a small incline of the printed micromodel during the X-ray scanning sequence, as well as the observed divergence of two micromodels (i.e. for $\lambda _s=35 \,\delta x$ and $45 \,\delta x$) from the targeted dimension of 5cm in height. The latter divergence is less than 3%. Therefore, the plotted orange curve corresponds to the equation;

15 $$\begin{aligned} \phi _{Xray}(z)+\Delta \bar{\phi }=\alpha (1-z/L) \end{aligned}$$

where $\phi _{Xray}(z)$ is the x-y averaged value of porosity, as measured using X-ray imaging, *z* is the depth in lattice units and $L=500\delta x$ is the height of the micromodel. The re-scaling parameter $\alpha$ takes a different value for each domain.

**Table 2:** Numerical calculation of the permeability of the cubic and their corresponding cylindrical digital domains using the LB method.

	Cubic design		Cylindrical design		
Correlation length, $\lambda _s$ ($\delta x$)	Porosity, $\phi$	Permeability, $K_z$ ($\delta x^2$)	Porosity, $\phi$	Permeability, $K_z$ ($\delta x^2$)	Permeability difference (%)
15	0.450	0.66	0.447	0.57	13
25	0.450	1.77	0.444	1.34	24
35	0.450	3.41	0.442	2.12	38
45	0.450	5.48	0.440	2.98	47

**Table 3:** Porosity and permeability of printed 3D micromodels. The LB permeability is measured numerically by applying the LB method on a digital reconstructions (obtained by XRCT imaging) of the actual printed samples, while the experimental measurements are obtained using the microfluidic setup of Fig. 14.

Correlation length, $\lambda _s$ [$\delta x$]	Porosity, $\phi$	LB permeability of printed micromodel $(m^2)$	Experimental permeability of printed micromodel $(m^2)$	Permeability difference [%]
15	0.335	$2.0\times {10^{-9}}$	$1.5\times {10^{-9}}$	25
25	0.360	$6.2\times {10^{-9}}$	$5.3\times {10^{-9}}$	14
35	0.400	$1.2\times {10^{-8}}$	$1.7\times {10^{-8}}$	41
45	0.400	$1.9\times {10^{-8}}$	−	−

We conclude this contribution by discussing the experimentally measured permeabilities of the 3D micromodels with the corresponding numerical results. Given that the physical 3D micromodels demonstrate an undershoot regarding the required porosity (that is however homogeneously distributed along the z-axis), we reconstruct the same digital designs with a porosity that matches exactly the one that was calculated using X-ray imaging. This is an important step in order to make a meaningful comparison with the experimental results, given also that the permeability was found to scale with the third power of the porosity (see Eq. 7). Table 3 shows a comparison between the experimentally measured permeability using the previously described microfluidic setup and the numerical LB calculation in domains with exactly the same porosity. Interestingly enough, the experimental permeability for the micromodels with $\lambda _s=15\delta x$ and $25\,\delta x$ is in good agreement with the numerical values. A large divergence in the order of $40\%$ is observed for larger values of $\lambda _s=35\,\delta x$, while an accurate measurement could not be accomplished for $\lambda _s=45\,\delta x$. This should be attributed to the typical pore size becoming comparable with the dimensions of our inlet tubing of the experimental setup. Thus the pressure drop within the inlet tubing becomes significantly higher than the one within the micromodel. This effect masks the overall pressure drop with the micromodel producing significant errors.

Additionally, the Reynolds numbers obtained from experimental and numerical investigations based on scanning domains fell within a very close range. For the experiments, Reynolds numbers ranged from $6.3 \times 10^{-6}$ to $3.135 \times 10^{-4}$, corresponding to samples with $\lambda$ ranging from 15 to 35 $\delta {x}$. In contrast, for the scanning domain, Reynolds numbers ranged from $8.63 \times 10^{-6}$ to $2.580 \times 10^{-4}$, corresponding to $\lambda$ ranging from 15 to 45 $\delta {x}$. It is worth noting that in the numerical simulations, Reynolds numbers varied from $2.637 \times 10^{-8}$ to $9.310 \times 10^{-5}$, covering both lower ($\phi =0.15, \lambda = 15\,\delta {x}$) to higher ($\phi =0.45, \lambda =45\,\delta {x}$) porosity and correlation lengths. These simulations were conducted within cubic domains. Based on this evaluation, we anticipate similar fluid behavior within the scaled-up domain as long as the Reynolds number is respected.

## Conclusions

In this study we proposed and validated an integrated methodology for the design and construction of 3D micromodels that are suitable for the study of transport processes in porous media in combination with XRCT imaging. The micromodels bear the pore-scale characteristics of sandstone and were constructed at a very fine resolution (i.e. $16 \,\upmu \hbox {m}$) using a state-of-the-art 3D printing infrastructure. Despite the fact that the constructed micromodels correspond to scaled-up replicas of natural porous rocks, we demonstrate that the correlation between porosity, mean pore size, and permeability is reproduced by applying the appropriate dimensionless numbers, such as the Reynolds number for single-phase flow. The REV- and pore-scale characteristics of both the original digital designs and the physical micromodels were recovered using a combination of XRCT and microfluidic measurements. The experimental results were then coupled with robust pore-scale flow and geostatistics models that allowed for the explicit calculation of the effects of the design parameters on the intrinsic permeability and the spatial correlation of the velocity profiles. Our numerical and experimental measurements revealed an excellent match between the properties of the designed and fabricated 3D domains, thus demonstrating the robustness of the proposed methodology for the construction of 3D REV micromodels with fine-tuned and well-controlled pore-scale characteristics.

We have also conducted a pore-scale numerical study over a wider range of 3D digital domain realizations to study the effects of the pore-scale parameters on the permeability at the REV-scale. Our study revealed a very good match between the measured values and the predictions of the Kozeny–Carman formulation using a single control parameter that is found to have a practically constant value for $\phi \ge 0.2$. At smaller values of the permeability, a significant increase in the fraction of the non-percolating pores was observed in the digital domains, leading to a divergence from the Kozeny–Carman formulation as the domain becomes non-percolating. A critical porosity value was subsequently determined based on these measurements.

Throughout this extensive investigation conducted in single-phase flow using these replicas, we have established a robust foundation for comprehensive further realistic pore-scale research. Our approach involves the use of meticulously designed and fabricated replicas, granting us complete control over porosity, pore sizes, and permeability. This approach also presents a unique opportunity to systematically explore the impact of pore-scale microstructure on REV-scale characteristics with reproducibility. In the context of XRCT, these enlarged pores, without compromising representativeness and realism regarding the porosity-permeability relationship when compared to natural rock, can enhance visibility. Building upon the foundation established in this study, we anticipate that this approach will facilitate extensive research in two-/multiphase flow and provide a platform for further investigations in this domain. To achieve this, it is essential to ensure that the Capillary number is respected within the domain, and further investigation of the sample wettability is warranted. Specifically, the use of enlarged yet still representative pores in our domain, without sacrificing dimensionality, allows for systematic variations in mean pore size and porosity. This, in turn, allows for effective visualization of scenarios such as the displacement of two different immiscible fluids while accurately tracking the movement of the interface within a realistic and well-validated domain.

## Data Availability

The image data of the scanned micromodels (projection datasets, reconstructed datasets including metadata, and binarized datasets) as well as the results of conducted numerical investigation that support the findings of this study have been deposited in the Data Repository of the University of Stuttgart (DaRUS) and can be accessed through the corresponding Refs.^71,72^.
